# Granulomatous and Sarcoid-like Immune-Related Adverse Events following CTLA4 and PD1 Blockade Adjuvant Therapy of Melanoma: A Combined Analysis of ECOG-ACRIN E1609 and SWOG S1404 Phase III Trials and a Literature Review

**DOI:** 10.3390/cancers15092561

**Published:** 2023-04-29

**Authors:** Islam Eljilany, Arish Noor, Mahati Paravathaneni, Ibrahim Yassine, Sandra J. Lee, Megan Othus, James Moon, John M. Kirkwood, Vernon K. Sondak, Antoni Ribas, Kenneth F. Grossmann, Ahmad A. Tarhini

**Affiliations:** 1 Houston Lee Moffitt Cancer Center and Research Institute, Tampa, FL 33612, USA; 2 Department of Psychology, College of Life Sciences, University of California, Los Angeles, CA 90095, USA; 3 Dana Farber Cancer Institute, Harvard Medical School, Boston, MA 02215, USA; 4 Fred Hutchinson Cancer Research Center, Seattle, WA 98109, USA; 5 UPMC Hillman Cancer Center, University of Pittsburgh, Pittsburgh, PA 15213, USA; 6 Merck and Co., Kenilworth, NJ 07033, USA

**Keywords:** adjuvant melanoma, granulomatous lesions, interferon, ipilimumab, pembrolizumab, sarcoid-like lesions

## Abstract

**Simple Summary:**

Immune checkpoint inhibitors (ICIs) can cause granulomatous and sarcoid-like lesions (GSLs) in several organs, and limited data existing that adequately inform the incidence and severity. This study examined GSL incidence in patients with high-risk melanoma treated with CTLA4 or PD1 blocking adjuvant therapy in two randomized clinical trials, the ECOG-ACRIN E1609 and SWOG S1404. The investigation identified 11 GSL cases among 2878 patients treated with ICIs or high-dose IFNα-2b. Grade III events were dominantly reported. Lung, mediastinal lymph nodes, skin, subcutaneous tissue, and eye were implicated in the reported GSLs. In addition, 62 literature reports were summarized. In conclusion, GSLs following anti-CTLA4 or anti-PD1 adjuvant treatment were uncommonly reported.

**Abstract:**

Background: Treatment with immune checkpoint inhibitors (ICIs) has been linked to granulomatous and sarcoid-like lesions (GSLs) affecting different organs. This study sought to evaluate GSL incidence in patients with high-risk melanoma treated with cytotoxic T-lymphocyte antigen 4 (CTLA4) or programmed cell death 1 (PD1) blockade adjuvant therapy in two clinical trials: ECOG-ACRIN E1609 and SWOG S1404. Descriptions and GSL severity ratings were recorded. Methods: Data were collected from ECOG-ACRIN E1609 and SWOG S1404. Descriptive statistics along with GSL severity grades were reported. Additionally, a literature review for such cases was summarized. Results: A total of 11 GSL cases were reported among 2878 patients treated with either ICI or with High-Dose Interferon Alfa-2b (HDI) in ECOG-ACRIN E1609 and SWOG S1404 trials. Cases were numerically more commonly reported with ipi10, followed by pembrolizumab, ipi3, and HDI, respectively. Most of the cases were grade III. Further, organs involved included lung, mediastinal lymph nodes, skin and subcutaneous tissue, and eye. Furthermore, a summary of 62 reports in the literature was described. Conclusions: GSLs following anti-CTLA4 and anti-PD1 antibody therapy in patients with melanoma were reported unusually. Reported cases ranged in grade from I to III and appeared manageable. Careful attention to these events and their reporting will be essential to better guide practice and management guidelines.

## 1. Introduction

In recent years, the advent of immunotherapy has changed the landscape of cancer treatment. Contrary to the previous standard of care (chemotherapy), the cytotoxic T-lymphocyte antigen 4 (CTLA-4) and programmed cell death 1 (PD-1) immune checkpoint inhibitors (ICIs) offer relatively well-tolerated and effective regimens that have improved survival outcomes across various malignancies including melanoma [1,2,3,4]. These advances in survival were also seen in the melanoma adjuvant setting where, initially, the CTLA-4 inhibitor ipilimumab, 3 or 10 mg/kg (ipi3 or ipi10, respectively) (Yervoy^®^, Bristol-Myers Squibb, New York, NY, USA), and later the PD-1 inhibitors pembrolizumab (Keytruda^®^, Merck & Co., Rahway, NJ, USA) and nivolumab (Opdivo^®^, Bristol-Myers Squibb, New York, NY, USA) demonstrated significant benefits and have become the current standard of care [5].

ICIs act by enhancing our natural immune system and overcoming physiologic immune regulation mechanisms. Blocking CTLA-4, PD-1, or its partner ligand 1 (PD-L1), leads to enhancing the activation and increasing the proliferation of effector T cells [6]. As might be expected, adverse reactions resulting from ICIs are autoimmune and can affect various organs, including skin, bowel, eyes, lymph nodes, endocrine glands, liver, lungs, and kidneys, amongst others. Some of the lesser-known and underreported immune-related adverse events (irAEs) are granulomatous and sarcoid-like lesions (GSLs), which are occasionally observed in the clinic but tend to occur late in the course of treatment and can be asymptomatic [7]. GSL lesions are usually asymptomatic but may cause cough or dyspnea in thoracic GSLs. Thoracic GSLs are commonly accidentally seen as nodular ground-glass lung opacities on systemic radiologic imaging investigations and may be misinterpreted for melanoma development, leading to premature immune treatment withdrawal. Thus, biopsies must confirm the diagnosis. Non-caseating granulomas support GSL [8]. Like sarcoidosis, GSLs can appear in tattoos and scars as papules, plaques, and nodules [9].

The underlying pathogenesis of ICI-associated GSLs appears to be similar to sarcoidosis. It is expected to involve inflammation resulting from the release of pro-inflammatory cytokines, such as interferon-γ (IFN-γ), interleukin (IL)-2, IL-12, IL-18, and tumor necrosis factor-alpha (TNF-α). Systemic inflammation may lead to the activation and aggregation of macrophages and granuloma formation. After that, macrophages further recruit TH17 cells resulting in the secretion of IL17, which further matures formed granulomas [10]. Drugs-like ipilimumab that acts through the CTLA-4 blockade promotes TH17 cell proliferation and the secretion of IL17 and pro-inflammatory IL-6 and TNF-α cytokines [11]. The PD1/PD-L1 pathway is similarly involved in regulating the balance of regulatory T cells and TH17 cells, and their inhibition results in TH17 cell hyperactivity and IL-17 hypersecretion [12,13]. Through this pathway, anti-PD1 antibodies have been reported to exacerbate underlying diseases, such as tuberculosis and sarcoidosis [14].

Our study related to GSLs is nested within two previously reported phase III randomized controlled trials (RCTs) that tested adjuvant ICIs in high-risk melanoma [15,16]. The first trial is ECOG-ACRIN E1609, a Phase III Study of Adjuvant Ipilimumab (3 or 10 mg/kg) Versus High-Dose Interferon Alfa-2b (HDI) for Resected High-Risk Melanoma: North American Intergroup E1609 [15]. The other study is SWOG S1404, a Phase III Randomized Trial of Physician/Patient Choice of Either High-dose Interferon or Ipilimumab to MK-3475 (Pembrolizumab) in Patients with High-risk Resected Melanoma [16]. The E1609 study reported that ipi3 and ipi10 showed high incidence rates of immune-related toxicity, defined as a variety of adverse events that are considered consistent with ICIs that are grade 3 or higher, with 28.5% and 46.3% for IPI3 and IPI10, respectively [16]. At the same time, the SWOG S1404 trial found that an average of 17% and 43% of patients treated with pembrolizumab or ipi10, respectively had experienced adverse events of grade 3 or higher that were directly attributed to the treatment [17]. Apart from both trials’ interesting findings, they did not examine the incidence of GSLs among patients with melanoma given their rarity. Additionally, while there are sporadic case reports and limited case series that report GSLs with ICI use, the actual incidence remains unknown, including the incidence from the organs involved [17]. Therefore, this study aims to estimate the incidence of GSLs following adjuvant therapy with ipilimumab (ipi3 and ipi10), pembrolizumab, and HDI in patients with high-risk melanoma treated in the context of the ECOG-ACRIN E1609 and SWOG S1404 adjuvant clinical trials. Furthermore, we provide a comprehensive summary of 62 reports of GSLs in the literature.

## 2. Materials and Methods

### 2.1. Study Design and Data Collection

The study’s design was descriptive. Collected AEs were secondary data that were utilized from the two clinical trials. Briefly, ECOG-ACRIN E1609 tested adjuvant immunotherapy with ipi3 or ipi10 versus HDI in 1670 patients with high-risk resected melanoma [15]. Similarly, SWOG S1404 investigated pembrolizumab versus the investigator/patient choice of ipi10 or HDI in 1207 patients with the same condition [16].

Both studies’ protocols were approved by each participating institution’s institutional review board (IRB) of record and conducted in compliance with Good Clinical Practice (GCP) principles as outlined by the International Conference on Harmonization (ICH).

Data collected included GSL events, organs affected by GSLs, and the reported grade of the AE in the treatment arms of ipi3, ipi10, pembrolizumab, and HDI. Grading of the severity of irAEs was based on the Common Terminology Criteria for Adverse Events (CTCAE) V.5 criteria. Cases treated with the same adjuvant regimen on ECOG-ACRIN E1609 and SWOG S1404 were combined, such as ipi10 and HDI. Descriptive statistics were applied to calculate the GSL incidence.

### 2.2. Literature Review

A comprehensive literature review was carried out utilizing PubMed, using the following index terms in search (“sarcoidosis” OR “Sarcoid-like” OR “granulomatosis”) AND (“ipilimumab” OR “high dose interferon” OR “HDI” OR “pembrolizumab” OR “Anti-CTLA-4” OR “anti-PD-1” OR “checkpoint inhibitors” OR “immune checkpoint” OR “melanoma treatment”) to identify published reported cases with GSL in patients with melanoma taking either ICIs or HDI.

## 3. Results

Collectively there were 2878 patients treated with ICIs or with HDI in the ECOG-ACRIN E1609 and SWOG S1404 clinical trials. Out of them, a total of 932 patients received treatment with ipi10, 783 with HDI, 640 with pembrolizumab, and 523 with ipi3. Overall, 11 cases of GSLs were reported amongst these 2878 patients, as shown in Table 1.

A closer inspection of the table shows that ipi10 had the highest incidence of reported events (0.64%), followed by pembrolizumab (0.47%), then 0.19% and 0.13% for ipi3 and HDI, respectively. The most affected organs were the lungs and mediastinal lymph nodes (n = 8) (reported as sarcoidosis, granulomatous inflammation, noncaseating granulomatous lymphadenitis), followed by two events affecting the skin and subcutaneous tissue (granuloma annulare, granulomatous dermatitis) and, finally, one event involving the eye (ocular sarcoidosis). The severity of GSLs ranged from grade I to III, including five events with grade III, followed by four and two cases with grade I and grade II, respectively. As observed in Table 1, one case of grade III was reported with ipi3 and another with HDI. Six cases registered with ipi10 are as follows: two with grade III, two with grade II, and two with grade I

Most cases were managed with observation. In one case of pulmonary sarcoidosis in a patient treated with pembrolizumab, the patient was initially diagnosed with asymptomatic sarcoidosis reported as grade 1, at week 30 from treatment initiation. Pembrolizumab was continued per protocol, and the patient was placed under close surveillance by a local pulmonologist. The patient later received week 34 treatment. At that time, the patient was still asymptomatic. However, a week later, the patient began experiencing dyspnea, cough with a burning sensation in lungs, fatigue, and joint pain. Symptoms subsequently improved or mostly resolved in about one week. A follow-up chest X-ray as part of the pulmonologist’s surveillance as well as a routine PET-CT scan per protocol were later completed. The chest X-ray demonstrated bilateral pulmonary infiltrates. The PET-CT demonstrated interval development of extensive patchy bilateral peripheral airspace infiltrates. The decision was made to discontinue protocol treatment and start corticosteroids. The toxicity grade was upgraded to grade 3.

The literature review uncovered that there are approximately 62 published case reports of GSL in the context of ICIs in patients with melanoma. Importantly, we conducted a careful review of the case reports and do not believe there is overlap between any of the literature reports and the cases reported within E1609 and S1404. The earliest report was in 2009 by Eckert et al., who reported one case of developed cutaneous, pulmonary, mediastinal lymphadenopathy GSLs with anti-CTLA4, while the latest one was reported by Marcoval et al. in 2021 about a patient who developed cutaneous GSL upon using anti-PD1. Supplemental Table S1 provides a summary of the number of cases reported in each report, along with the organs involved and the ICI agent used to treat melanoma. Overall, there were 50 GSL cases reported with anti-PD1 as monotherapy, 35 cases with anti-CTLA4 as monotherapy, and 21 cases with anti-PD1 in combination with anti-CTLA4. The most reported GSL type was lymphatic (n = 60), and after that, cutaneous (n = 48), then pulmonary (n = 47), and, lastly, ocular (n = 4) and neurological (n = 3), respectively (Table 2).

## 4. Discussion

The broad term of GSLs includes granulomatous lesions, such as granuloma annulare, granulomatous dermatitis, granulomatous panniculitis, and various other granulomatous reactions. Although the lesions are characterized histologically by non-caseating granulomas, these lesions differ from sarcoidosis in that sarcoidosis is a systemic multiorgan disease of unclear etiology resulting in non-caseating granuloma formation. GSLs, as termed here, develop in the absence of a prior diagnosis of systemic sarcoidosis and occur as a result of ICI therapy. Similar to other irAEs, GSLs result from enhanced immune activation [18,19]. GSL appears to be one of the irAEs that is infrequently found in the clinic as it tends to be asymptomatic and can develop late in the therapy cycle [7].

Prior case reports documented such cases; however, there is no clear theory about the possible incidence of GSLs among different ICI users with melanoma [17]. The current work aimed to define the possible incidence of GSLs among patients with melanoma who are being treated with ICIs. Consequently, this study determined the number of developed GSL cases in such participants in the context of the ECOG-ACRIN E1609 and SWOG S1404 phase III trials [15,16]. The most obvious finding to emerge from the results is that the incidence of GSLs among patients with high-risk melanoma was very low (0.38%). This percentage is much lower than that reported last year by Melin et al. [20], who reported that ICI-induced GSLs were 5% in patients with melanoma, as estimated from a single-center retrospective observational study. This inconsistency may be due to the differences in design and the sample size examined.

What is curious about the current results is that more than half of the discovered GSL cases came from the higher dose of ipi10, relative to one event with a lower dose of ipi3. Using a higher dose of ipilimumab also resulted in a greater number of involved organs, suggesting a dose-dependent component to the potential of developing GSLs with ipilimumab. The pembrolizumab-related incidence of GSLs was also uncommon and was in between that of ipi3 and ipi10. Additionally, the findings of the current literature review result in a higher number of GSLs with anti-PD1 than anti-CTLA4, which supports the previous review by Cornejo et al. [21], which stated that PD1/PD-L1 blockade accounted for a higher incidence of GSLs ((59.3%) 35 of 59) relative to CTLA-4 blockade ((33.9%) 20 of 59). Adding to that, in our study, HDI usage resulted in the lowest incidence of GSLs (0.13%), in comparison to ICIs. A likely explanation might be that HDI acts through exposure to INF-α, while anti-CTLA4 and anti-PD1 inhibition result in activation through multiple pathways and cytokines, resulting in a more robust immune activation, as described above. HDI-associated lesions have been reported to respond to holding treatment and the utilization of systemic corticosteroids [22].

Another important finding in our study is that the most affected organs were the lung and mediastinal lymph nodes, followed by the skin and subcutaneous tissue and the eye. These results are consistent with the current literature, reporting the highest incidence in thoracic and mediastinal lymph nodes (92.9%), followed by the lungs (50%) and skin (10.7%) [8,23]. Other less frequently involved organs include the spleen and subepithelial surface granulomatosis observed in the colon [24,25,26].

GSL events in our study ranged in severity from grades I to III. Interestingly, grade III events accounted for the highest proportion of the reported GSL events. This is likely due to the fact that our study population consisted of patients with high-risk melanoma treated in the adjuvant setting, where the incidence of irAEs has been shown to be higher than those with more advanced metastatic melanoma. In contrast, Cabanie’s study included patients with various advanced malignancies [8].

As mentioned in the Results section, our literature review showed that melanoma appears to have a higher incidence of GSLs relative to other malignancies. Apalla et al. [27] reported that 81.3% of ICI-mediated GSL lesions were reported in patients with melanoma. This might be attributed to the high levels of circulating TH17 cells and IL17 that have been reported in patients with melanoma prior to the initiation of immunotherapy and could be contributing to the increased risk of developing GSL [28,29]. Unfortunately, the majority of the literature review reported in Supplementary Table S1 did not document the total number of patients per regimen to calculate the incidence of GSL.

Notably, most GSL cases are improved or resolved by corticosteroids. Steroids use for GSL management varies in the literature. For example, 57% of Cornejo et al.’s [21] patients took systemic corticosteroids, and 49% had immunotherapy dosage holding or cessation, while 17.9%–41% of individuals used systemic corticosteroids in other trials [9,30]. The absence of GSL management recommendations and clinical discretion may explain the diversity in therapy. IFN-α2a and IFN-α2b have been used to treat melanoma systemically [31]. They have also been used for chronic hepatitis, melanoma, renal cell carcinoma, and hematological malignancies [22,32,33].

The Society for Immunotherapy of Cancer (SITC) Toxicity Management Working Group’s consensus guidelines for managing ICI-associated GSLs are helpful [34]. Holding ICI treats grade I sarcoidosis; while prednisone at 1 mg/kg is suggested for grade II or higher related GSLs with increasing radiographic changes, substantial respiratory symptoms, decrease in lung function, the involvement of essential extrapulmonary organs, or hypercalcemia [34]. Future SITC irAE management guidelines could examine GSL types and give more toxicity-specific treatment advice.

The uniqueness of this study is that it has the largest cohort of patients with melanoma evaluated for the incidence of ICI-induced GSLs. Furthermore, it compared the incidence across different types of therapies, including anti-PD1, anti-CTLA-4, and HDI, while also comparing different dose strengths of ipilimumab. Not only that, but, the data were collected from well-conducted RCTs, which minimizes possible confounding.

Our study has some limitations that could temper our findings. Firstly, the collected data were secondary data, and it primarily explored GSLs in patients with melanoma and did not provide information on GSL incidence in other malignancies. Given the challenges with cross-trial comparisons, a comparison between anti-PD-1 and anti-CTLA-4 could not be drawn.

Finally, GSL cases might likely be underrecognized and underreported overall. With regard to the E1609 and S1404 phase 3 trials, both of these studies were adjuvant studies where progression or relapse was defined by disease recurrence that was required to be histologically confirmed as part of the study protocols. Therefore, it is unlikely that a GSL case was assumed to be a treatment failure without biopsy. On the other hand, radiologic imaging studies that were required for the study were not centrally reviewed. Therefore, in the absence of symptoms, radiologic changes that may have been designated as “nonspecific” or “likely inflammatory” may have been ignored. One key question relates to the long-term implications of such nonspecific findings and whether these may evolve years later. This is a question that we hope to obtain some answers to in the coming years. These studies have long-term follow-up plans built into the protocols, including up to 20 years in E1609, with the intent to capture late-arising and rare adverse events. Similarly, it would be important to follow patients longitudinally for such findings in clinical practice and report evolving cases in order to inform the field better and serve our patients.

## 5. Conclusions

This study set out to determine the incidence of GSLs among patients with high-risk melanoma taking ICIs. The findings clearly indicate that they are possibly uncommon. Since most patients are asymptomatic, GSLs are likely underrecognized and underreported. Therefore, continued assessment of these lesions and their reporting is essential to guide practice and management guidelines; also, better toxicity-specific management recommendations are needed and should be included in future guidelines.

## Figures and Tables

**Table 1:** Granulomatous and sarcoid-like lesions immune-related adverse events reported in the ECOG-ACRIN E1609 and SWOG S1404 clinical trials.

Clinical Trial	Organ(s) Involved	Ipi3 (n = 523)	Ipi10 (n = 932)	Total Ipi (n = 1455)	HDI (n = 783)	Pembrolizumab (n = 640)
ECOG-ACRIN E1609	Ocular	0	1 (Gr 2)	1	0	NA
	Skin	0	1 (Gr 1)	1	0	NA
	Lung and Lymphatic	1 (Gr 3)	2 (Gr 3)	3	1 (Gr 3)	NA
SWOG S1404	Skin	NA	1 (Gr 1)	1	0	0
	Lymphatic	NA	0	0	0	1 (Gr 1)
	Lung and Lymphatic	NA	1 (Gr 2)	1	0	2 (Gr 1, Gr 3)
Total, n (%) 95% C.I		1 (0.19%) 0.003–1.08%	6 (0.64%) 0.3–1.4%	7 (0.48%) 0.23–0.99%	1 (0.13%) 0.02–0.72%)	3 (0.47%) 0.16–1.37%

ECOG-ACRIN E1609: Phase III Study of Adjuvant Ipilimumab (3 or 10 mg/kg) Versus High-Dose Interferon Alfa-2b for Resected High-Risk Melanoma: North American Intergroup E1609. SWOG S1404: Phase III randomized trial of physician/patient choice of either high dose Interferon or Ipilimumab to MK-3475 (Pembrolizumab) in patients with high-risk resected Melanoma. Abbreviations: Gr, severity grades I-III; HDI; high-dose IFN-α; Ipi3; ipilimumab 3 mg/kg, Ipi10; ipilimumab 10 mg/kg.

**Table 2:** The number of granulomatous and sarcoid-like lesions per the organ systems involved in patients with melanoma treated with immune checkpoint inhibitor as reported in our literature review.

Organ Involved *	Immune Checkpoint Inhibitor		
	PD1	CTLA4	PD1 + CTLA4
Cutaneous, no.	25	13	10
Pulmonary, no.	23	21	3
Lymphatic, no.	26	23	11
Ocular, no.	4	0	0
Neurologic, no.	0	1	2
Other visceral, non-visceral, no.	2 ^¥^	4 ^#^	0

* Single organ systems counted even if a patient presented with more than one organ involvement as listed in Supplemental Table S1. ^¥^ Bone, parotid gland. ^#^ Spleen. Abbreviations: CTLA4; cytotoxic T-lymphocyte antigen 4, PD1; programmed cell death 1.

## Data Availability

The datasets used and/or analyzed during the current study are available from the corresponding authors upon a reasonable request.

## Supplementary Materials

The following supporting information can be downloaded at: https://www.mdpi.com/article/10.3390/cancers15092561/s1, Table S1: Reports of granulomatous and sarcoid-like lesions in the context of immune checkpoint inhibitors in patients with melanoma [17,24,26,27,28,30,35,36,37,38,39,40,41,42,43,44,45,46,47,48,49,50,51,52,53,54,55,56,57,58,59,60,61,62,63,64,65,66,67,68,69,70,71,72,73,74,75,76,77,78,79,80,81,82,83,84,85,86,87,88,89,90].
