# Supplementary material for: Granulomatous and Sarcoid-like Immune-Related Adverse Events following CTLA4 and PD1 Blockade Adjuvant Therapy of Melanoma: A Combined Analysis of ECOG-ACRIN E1609 and SWOG S1404 Phase III Trials and a Literature Review
Source: Cancers (Basel). 2023 Apr 29;15(9):2561. doi: 10.3390/cancers15092561 (PMC10177189; doi:10.3390/cancers15092561)
Supplement: Supplementary file 1 [file cancers-15-02561-s001.zip › cancers-2309066-supplementary.pdf]

Table S1. Reports of granulomatous and sarcoid-like lesions in the context of immune checkpoint inhibitors in patients with melanoma.

Author	Number of cases	Type of events	ICI	Total number of patients treated	Incidence (%) (95% C.I)	Reference
(Dimitriou, Frauchiger et al. 2018)	1	Mediastinal/ hilar lymphadenopathy	PD-1	N/A	N/A	[35]
	1	Cutaneous	PD-1	N/A	N/A	
	2	Cutaneous, mediastinal/ hilar lymphadenopathy	PD-1	N/A	N/A	
(Tetzlaff, Nelson et al. 2018)	1	Cutaneous, mediastinal/ hilar lymphadenopathy	CTLA-4	N/A	N/A	[30]
	1	Cutaneous, mediastinal/ hilar lymphadenopathy	PD-1	N/A	N/A	
	1	Mediastinal/ hilar lymphadenopathy	PD-1	N/A	N/A	
(Le Burel, Champiat et al. 2017)	1	Ocular, Cutaneous, pulmonary, lymphadenopathy	PD-1	N/A	N/A	[36]
(Lomax, McGuire et al. 2017)	1	Pulmonary, cutaneous, lymphadenopathy	PD-1	N/A	N/A	[28]
	1	Cutaneous, lymphadenopathy	PD-1	N/A	N/A	
	1	Pulmonary	PD-1	N/A	N/A	
(Reddy, Possick et al. 2017)	2	Cutaneous, pulmonary	CTLA-4 and PD-1	N/A	N/A	[37]
(Toumeh, Sakhi et al. 2016)	1	Diffuse Lymphadenopathy	CTLA-4	N/A	N/A	[38]
(Yatim, Mateus et al. 2018)	1	Cutaneous, pulmonary	PD-1	N/A	N/A	[39]
(Firwana, Ravilla et al. 2017)	1	Cutaneous, mediastinal/ hilar lymphadenopathy	CTLA-4	N/A	N/A	[40]
	1	Diffuse lymphadenopathy, spleen	CTLA-4	N/A	N/A	
	1	Cutaneous, pulmonary, diffuse lymphadenopathy	PD-1	N/A	N/A	
(Danlos, Pages et al. 2016)	1	Cutaneous, mediastinal lymphadenopathy	PD-1	N/A	N/A	[17]
(Martinez Leborans, Esteve Martinez et al. 2016)	1	Cutaneous	CTLA-4	N/A	N/A	[41]
(Tissot, Carsin et al. 2013)	1	Cutaneous, pulmonary, mediastinal lymphadenopathy	CTLA-4	N/A	N/A	[42]
(Eckert, Schoeffler et al. 2009)	1	Cutaneous, pulmonary, mediastinal lymphadenopathy	CTLA-4	N/A	N/A	[43]

(Seve, Schott et al. 2009)	1	pulmonary	CTLA-4	N/A	N/A	[44]
(Reule and North 2013)	1	Cutaneous, pulmonary	CTLA-4	N/A	N/A	[45]
(McKenna, Molloy et al. 2018)	1	Cutaneous, pulmonary, diffuse lymphadenopathy	PD-1	N/A	N/A	[46]
(Jespersen, Bjursten et al. 2018)	1	Cutaneous, mediastinal lymphadenopathy	PD-1	N/A	N/A	[47]
(Burillo-Martinez, Morales-Raya et al. 2017)	1	Cutaneous, pulmonary	PD-1	N/A	N/A	[48]
	1	Cutaneous (Granulomatous panniculitis)	PD-1	N/A	N/A	
(Andersen, Norgaard et al. 2014)	1	Spleen	CTLA-4	N/A	N/A	[26]
(Balestra, Benzaquen et al. 2017)	1	Pulmonary, mediastinal lymphadenopathy	PD-1	N/A	N/A	[49]
(Cheshire, Board et al. 2018) check	2	Mediastinal/ hilar lymphadenopathy	PD-1	N/A	N/A	[50]
	1	Cutaneous, mediastinal/ hilar lymphadenopathy	PD-1	N/A	N/A	
(Wesselius, DeLeon et al. 2018)	1	Lymphadenopathy	PD-1	N/A	N/A	[51]
(Gutzmer, Koop et al. 2017)	1	Pulmonary	PD-1	N/A	N/A	[52]
(Lidar, Giat et al. 2018)	2	Pulmonary	PD-1	12	16.66 (4.70- 44.80)	[53]
(Al-Dliw, Megri et al. 2017)	1	Pulmonary	PD-1	N/A	N/A	[54]
(Nandavaram and Nadkarni 2018)	1	Mediastinal/ hilar lymphadenopathy	CTLA-4	N/A	N/A	[55]
(Wilgenhof, Morlion et al. 2012)	1	Pulmonary, diffuse lymphadenopathy, spleen	CTLA-4	N/A	N/A	[56]
(Murphy, Kennedy et al. 2014)	1	Mediastinal/ hilar lymphadenopathy, CNS	CTLA-4	N/A	N/A	[57]
(Berthod, Lazor et al. 2012)	1	Pulmonary	CTLA-4	N/A	N/A	[58]
(Vogel, Guislain et al. 2012)	1	Mediastinal/ hilar lymphadenopathy	CTLA-4	N/A	N/A	[59]
(Montaudie, Pradelli et al. 2017)	1	Pulmonary, Parotid gland	PD-1	N/A	N/A	[60]
(Koelzer, Rothschild et al. 2016)	1	Pulmonary	PD-1	N/A	N/A	[61]
(Bronstein, Ng et al. 2011)	8	Pulmonary, diffuse lymphadenopathy	CTLA-4	119	6.72 (3.45- 12.71)	[62]
(Gilardi, Colandrea et al. 2014)	1	Mediastinal, hilar lymphadenopathy, rectus abdominus muscle	CTLA-4	N/A	N/A	[63]
(Arellano, Mosley et al. 2018)	1	Diffuse lymphadenopathy	CTLA-4	N/A	N/A	[64]
(Brahmer, Tykodi et al. 2012)	1	Sarcoidosis (not otherwise specified)	PD-1	N/A	N/A	[65]
(Jiang, Patino et al. 2018)	1	Cutaneous	PD-1	N/A	N/A	[66]

(Tetzlaff, Jazaeri et al. 2017)	2	Cutaneous	CTLA 4 and PD-1	N/A	N/A	[67]
(Wu, Kwong et al. 2018)	3	Cutaneous	PD-1	N/A	N/A	[68]
(Kubicki, Welborn et al. 2018)	3	Cutaneous	CTLA-4	N/A	N/A	[69]
(Perret, Josselin et al. 2017)	2	Cutaneous	CTLA-4	N/A	N/A	[70]
(Diaz-Perez, Beveridge et al. 2018)	1	Cutaneous	CTLA-4	N/A	N/A	[71]
(Chorti, Kanaki et al. 2020)	8	Mediastinal/hilar lymphadenopathy	PD-1 and CTLA-4	45	17.78 (9.29- 31.33)	[72]
	1	Cutaneous, pulmonary	PD-1 and CTLA-4	45	2.22 (0.39- 11.57)	
	1	Cutaneous	PD-1 and CTLA-4	45	2.22 (0.39- 11.57)	
(Mobini, Dhillon et al. 2019)	2	Cutaneous	PD-1, CTLA-4	N/A	N/A	[73]
(Reuss, Kunk et al. 2016)	1	Hilar, subcarinal lymphadenopathy, cutaneous	PD-1 and CTLA-4	N/A	N/A	[74]
(Rodriguez, Lipson et al. 2019)	3	Mediastinal/hilar lymphadenopathy	PD-1	N/A	N/A	[75]
	1	Mediastinal/ hilar lymphadenopathy	CTLA -4	N/A	N/A	
	1	Mediastinal/ hilar lymphadenopathy	PD-1 and CTLA-4	N/A	N/A	
Laroche (Laroche, Alarcon Chinchilla et al. 2018)	1	Cutaneous, Pulmonary	PD-1	N/A	N/A	[76]
(Tan, Malinzak et al. 2018)	1	Neuro-sarcoidosis, mediastinal, hilar lymphadenopathy	PD-1 and CTLA-4	N/A	N/A	[77]
(Dunn-Pirio, Shah et al. 2018)	1	Neuro-sarcoidosis	PD-1 and CTLA-4	N/A	N/A	[78]
(Ung and Gragoudas 2020)	1	Mediastinal/hilar lymphadenopathy, lung, eye	PD-1	N/A	N/A	[24]
(Tulbah, Rowe et al. 2019)	1	Mediastinal/hilar lymphadenopathy, bone	PD-1	N/A	N/A	[79]
(Fukuchi, Hikawa et al. 2019)	1	Pulmonary, Mediastinal/hilar lymphadenopathy, eye	PD-1	N/A	N/A	[80]
(Lise and Audrey 2017)	1	Mediastinal/hilar lymphadenopathy, lung, eye	PD-1	N/A	N/A	[81]
(Nishino, Sholl et al. 2018)	4	lung	PD-1	N/A	N/A	[82]
(Smith, Mitchell et al. 2018)	1	Mediastinal/hilar lymphadenopathy, lung, cutaneous	PD-1	N/A	N/A	[83]

(Wang, Patel et al. 2018)	1	Cutaneous	PD-1	N/A	N/A	[84]
(Woodbeck, Metelitsa et al. 2018)	1	Cutaneous	PD-1	N/A	N/A	[85]
(Lu 2019)	1	Mediastinal/hilar lymphadenopathy, cutaneous	PD-1	N/A	N/A	[86]
(Marcoval, Bauer-Alonso et al. 2021)	1	Cutaneous	PD-1	N/A	N/A	[87]
(Garanzini, Scaramuzza et al. 2020)	1	Mediastinal/hilar lymphadenopathy, lung, spleen	CTLA-4	N/A	N/A	[88]
(Cervantes, Rosen et al. 2019)	1	Cutaneous	CTLA-4	N/A	N/A	[89]
(Apalla, Kemanetzi et al. 2021)	1	Cutaneous	PD-1 and CTLA-4	N/A	N/A	[27]
(Yasin, Yadala et al. 2021)	1	Hilar/ mediastinal lymphadenopathy	CTLA-4	N/A	N/A	[90]

Abbreviations: CTLA4; cytotoxic T-lymphocyte antigen 4, ICI; immune checkpoint inhibitor, PD1; programmed cell death 1

3

References

1. Dimitriou, F.; Frauchiger, A.L.; Urosevic-Maiwald, M.; Naegeli, M.C.; Goldinger, S.M.; Barysch, M.; Franzen, D.; Kamarachev, J.; Braun, R.; Dummer, R.; Mangana, J.. Sar-coid-like reactions in patients receiving modern melanoma treatment. *Melanoma Res*. **2018**, *28(3)*, 230–236.
2. Le Burel, S.; Champiat, S.; Mateus, C.; Marabelle, A.; Michot, J.M.; Robert, C.; Belkhir, R.; Soria, J.C.; Lag-houati, S.; Voisin, A.L.,et al. Prevalence of im-mune-related systemic adverse events in patients treated with anti-Programmed cell Death 1/anti-Programmed cell Death-Ligand 1 agents: A single-centre pharmacovigi-lance database analysis. *Eur J Cancer*. **2017**,*82*,34–44.
3. Reddy, S.B.; Possick, J.D.; Kluger, H.M.; Galan, A.; Han, D. Sarcoidosis Following Anti-PD-1 and Anti-CTLA-4 Therapy for Metastatic Melanoma. *J. Immunother.* **2017**, *40*, 307–311, https://doi.org/10.1097/cji.0000000000000181.
4. Toumeh, A.; Sakhi, R.; Shah, S.; Arudra, S.K.; De Las Casas, L.E., Skeel, R.T. Ipilimumab-Induced Granuloma-tous Disease Occurring Simultaneously With Disease Progression in a Patient With Metastatic Melano-ma. *Am J Ther*. **2016**, *23(4)*,e1068–1071.
5. Yatim, N.; Mateus, C.; Charles, P. Sarcoidosis post-anti-PD-1 therapy, mimicking relapse of metastatic mela-noma in a patient undergoing complete remission. **2018**, *39*, 130–133, https://doi.org/10.1016/j.revmed.2017.11.008.
6. Firwana, B.; Ravilla, R.; Raval, M.; Hutchins, L.; Mahmoud, F.. Sarcoidosis-like syndrome and lymphade-nopathy due to checkpoint inhibitors. *J Oncol Pharm Pract*. **2017**, *23(8)*,620–624.
7. Martinez, L.L.; Esteve, M.A.; Victoria, M.A.M.; Alegre, d.M.V.; Berrocal, J.A. Cutaneous sarcoidosis in a mela-noma patient under Ipilimumab therapy. *Dermatol Ther*. **2016**,*29(5)*,306–308.
8. Tissot, C.; Carsin, A.; Freymond, N.; Pacheco, Y.; Devouassoux, G.; Naeije, R.; Chemla, D.; Dinh-Xuan, A.T.; Noordegraaf, A.V. Sarcoidosis complicating anti-cytotoxic T-lymphocyte-associated antigen-4 monoclonal antibody biotherapy. *Eur. Respir. J.* **2012**, *41*, 246–247, https://doi.org/10.1183/09031936.00107912.
9. Eckert, A.; Schoeffler, A.; Dalle, S.; Phan, A.; Kiakouama, L.; Thomas, L..Anti-CTLA4 monoclonal antibody in-duced sarcoidosis in a metastatic melanoma patient. *Dermatology*. **2009**, *218(1)*,69–70.
10. Seve, P.; Schott, A.M.; Pavic, M.; Broussolle, C.; Gilis, L.; Thomas, L. Sarcoidosis and Melanoma: A Referral Center Study of 1,199 Cases. *Dermatology* **2009**, *219*, 25–31, https://doi.org/10.1159/000213245.
11. Reule, R.B.; North, J.P. Cutaneous and pulmonary sarcoidosis-like reaction associated with ipili-mumab. *J Am Acad Dermatol*. **2013**, *69(5)*, e272–e273.
12. McKenna, M.C.; Molloy, K.; Crowther, S.; Feeney, J.; Gillis, A.; Connolly, M.; Kelleher, F. Pembrolizumab-Re-lated Sarcoid-Like Reaction Presenting as Reactivation of Quiescent Scars. *J. Oncol. Pr.* **2018**, *14*, 200–201, https://doi.org/10.1200/jop.2017.027383.
13. Jespersen, H.; Bjursten, S.; Ny, L.; Levin, M. Checkpoint inhibitor-induced sarcoid reaction mimicking bone metastases. *Lancet Oncol.* **2018**, *19*, e327, https://doi.org/10.1016/s1470-2045(18)30252-3.
14. Burillo-Martinez, S.; Morales-Raya, C.; Prieto-Barrios, M.; Rodriguez-Peralto, J.L.; Ortiz-Romero, P.L.. Pem-brolizumab-Induced Extensive Panniculitis and Nevus Regression: Two Novel Cutaneous Manifestations of the Post-immunotherapy Granulomatous Reactions Spectrum. *JAMA Dermatol*. **2017**, *153(7)*, 721–722.
15. Balestra, R.; Benzaquen, S.; Wang, J. Sarcoidosis-like Granulomatous Lung Reaction Associated with Anti–Programmed Death Receptor-1 Ligand Therapy. *Ann. Am. Thorac. Soc.* **2017**, *14*, 296–299, https://doi.org/10.1513/annalsats.201611-863le.
16. Cheshire, S.C.; Board, R.E.; Lewis, A.R.; Gudur, L.D.; Dobson, M.J. .Pembrolizumab-induced Sarcoid-like Reac-tions during Treatment of Metastatic Melanoma. *Radiology.* **2018**, *289(2)*, 564–567.
17. Wesselius, L.J.; DeLeon, T.T.; Gotway, M.B. A Sarcoidlike Reaction Mimicking Metastatic Malignancy in a Pa-tient With Melanoma Treated With Pembrolizumab. *Am. J. Roentgenol.* **2018**, *210*, W183–W184, https://doi.org/10.2214/ajr.17.19144.
18. Gutzmer, R.; Koop, A.; Meier, F.; Hassel, J.C.; Terheyden, P.; Zimmer, L.. Programmed cell death pro-tein-1 (PD-1) inhibitor therapy in patients with advanced melanoma and preexisting autoimmunity or ipilimumab-triggered autoimmunity. *Eur J Cancer*. **2017**, *75*, 24–32.
19. Lidar, M.; Giat, E.; Garelick, D.; Horowitz, Y.; Amital, H.; Steinberg-Silman, Y.. Rheumatic manifesta-tions among cancer patients treated with immune checkpoint inhibitors. *Autoimmun Rev*. **2018**, *17(3)*, 284–289.

20. Al-Dliw, M.; Megri, M.; Shahoub, I.; Sahay, G.; Limjoco, T.I.; Shweihat, Y.. Pembrolizumab reactivates pulmonary granulomatosis. *Respir Med Case Rep*. **2017**, *22*, 126–129.
21. Nandavaram, S.; Nadkarni, A. Ipilimumab-Induced Sarcoidosis and Thyroiditis. *Am. J. Ther.* **2018**, *25*, e379–e380, https://doi.org/10.1097/mjt.0000000000000545.
22. Wilgenhof, S.; Morlion, V.; Seghers, A.C.; Du Four, S.; Vanderlinden, E.; Hanon, S.. Sarcoidosis in a pa-tient with metastatic melanoma sequentially treated with anti-CTLA-4 monoclonal antibody and selec-tive BRAF inhibitor. *Anticancer Res*. **2012**,*32(4)*,1355–1359.
23. Murphy, K.P.; Kennedy, M.P.; Barry, J.E.; O'Regan, K.N.; Power, D.G.. New-onset mediastinal and central nervous system sarcoidosis in a patient with metastatic melanoma undergoing CTLA4 monoclonal an-tibody treatment. *Oncol Res Treat*. **2014**, *37(6)*,351–353.
24. Berthod, G.; Lazor, R.; Letovanec, I.; Romano, E.; Noirez, L.; Mazza-Stalder, J.; Speiser, D.; Peters, S.; Michielin, O. Pulmonary Sarcoid-Like Granulomatosis Induced by Ipilimumab. *J. Clin. Oncol.* **2012**, *30*, e156–e159, https://doi.org/10.1200/jco.2011.39.3298.
25. Vogel, W.V.; Guislain, A.; Kvistborg, P.; Schumacher, T.N.; Haanen, J.B.; Blank, C.U.. Ipilimumab-induced sarcoidosis in a patient with metastatic melanoma undergoing complete remission. *J Clin Oncol*. **2012**, *30(2)*, e7–e10.
26. Montaudie, H.; Pradelli, J.; Passeron, T.; Lacour, J.P.; Leroy, S.. Pulmonary sarcoid-like granulomatosis induced by nivolumab. *Br J Dermatol*. **2017**, *176(4)*, 1060–1063.
27. Koelzer, V.H.; Rothschild, S.I.; Zihler, D.; Wicki, A.; Willi, B.; Willi, N.. Systemic inflammation in a mela-noma patient treated with immune checkpoint inhibitors-an autopsy study. *J Immunother Cancer*. **2016**, *4*, 13.
28. Bronstein, Y.; Ng, C.S.; Hwu, P.; Hwu, W.-J. Radiologic Manifestations of Immune-Related Adverse Events in Patients With Metastatic Melanoma Undergoing Anti–CTLA-4 Antibody Therapy. *Am. J. Roentgenol.* **2011**, *197*, W992–W1000, https://doi.org/10.2214/ajr.10.6198.
29. Gilardi, L.; Colandrea, M.; Vassallo, S.; Travaini, L.L.; Paganelli, G.. Ipilimumab-induced immunomediated adverse events: possible pitfalls in (18)F-FDG PET/CT interpretation. *Clin Nucl Med*. **2014**, *39(5)*, 472–474.
30. Arellano, K.; Mosley, J.C.; Moore, D.C.. Case Report of Ipilimumab-Induced Diffuse, Nonnecrotizing Granulomatous Lymphadenitis and Granulomatous Vasculitis. *J Pharm Pract*. **2018**, *31(2)*, 227–229.
31. Brahmer, J.R.; Tykodi, S.S.; Chow, L.Q.; Hwu, W.J.; Topalian, S.L.; Hwu, P.. Safety and activity of anti-PD-L1 antibody in patients with advanced cancer. *N Engl J Med*. **2012**, *366(26)*, 2455–2465.
32. Jiang, B.; Patino, M.M.; Gross, A.J.; Leong, S.P.L.; Moretto, J.C.; Kashani-Sabet, M.. Diffuse granuloma-tous panniculitis associated with anti PD-1 antibody therapy. *JAAD Case Rep*. **2018**, *4(1)*, 13–16.
33. Tetzlaff, M.T.; Jazaeri, A.A.; Torres-Cabala, C.A.; Korivi, B.R.; Landon, G.A.; Nagarajan, P.; Choksi, A.; Chen, L.; Uemura, M.; Aung, P.P.; et al. Erythema nodosum-like panniculitis mimicking disease recurrence: A novel toxicity from immune checkpoint blockade therapy-Report of 2 patients. *J. Cutan. Pathol.* **2017**, *44*, 1080–1086, https://doi.org/10.1111/cup.13044.
34. Wu, J.; Kwong, B.; Martires, K.; Rieger, K.; Hung, S.-I.; Iyer, G.; Lacouture, M. Granuloma annulare associated with immune checkpoint inhibitors. *J. Eur. Acad. Dermatol. Venereol.* **2017**, *32*, e124–e126, https://doi.org/10.1111/jdv.14617.
35. Kubicki, S.L.; Welborn, M.E.; Garg, N.; Aung, P.; Patel, A.B. Granulomatous dermatitis associated with ipilimumab therapy (ipilimumab associated granulomatous dermatitis). *J. Cutan. Pathol.* **2018**, *45*, 636–638, https://doi.org/10.1111/cup.13267.
36. Perret, R.E.; Josselin, N.; Knol, A.-C.; Khammari, A.; Cassecuel, J.; Peuvrel, L.; Dreno, B.; Supported by GESTIM Nantes group of cutaneous adverse events induced by anticancer drugs Histopathological aspects of cutaneous erythematous-papular eruptions induced by immune checkpoint inhibitors for the treatment of metastatic melanoma. *Int. J. Dermatol.* **2017**, *56*, 527–533, https://doi.org/10.1111/ijd.13540.
37. Diaz-Perez, J.A.; Beveridge, M.G.; Victor, T.A.; Cibull, T.L. Granulomatous and lichenoid dermatitis after IgG4 anti-PD-1 monoclonal antibody therapy for advanced cancer. *J. Cutan. Pathol.* **2018**, *45*, 434–438, https://doi.org/10.1111/cup.13133.
38. Chorti, E.; Kanaki, T.; Zimmer, L.; Hadaschik, E.; Ugurel, S.; Gratsias, E.; Roesch, A.; Bonella, F.; Wessendorf, T.E.; Wälscher, J.; et al. Drug-induced sarcoidosis-like reaction in adjuvant immunotherapy: Increased rate and mimicker of metastasis. *Eur. J. Cancer* **2020**, *131*, 18–26, https://doi.org/10.1016/j.ejca.2020.02.024.
39. Mobini, N.; Dhillon, R.; Dickey, J.; Spoon, J.; Sadrolashrafi, K. Exclusive Cutaneous and Subcutaneous Sarcoidal Granulomatous Inflammation due to Immune Checkpoint Inhibitors: Report of Two Cases with

Unusual Manifestations and Review of the Literature.. *Case Rep. Dermatol. Med.* **2019**, *2019*, 6702870–7, https://doi.org/10.1155/2019/6702870.

40. Reuss, J.E.; Kunk, P.R.; Stowman, A.M.; Gru, A.A.; Slingluff, C.L.; Gaughan, E.M. Sarcoidosis in the setting of combination ipilimumab and nivolumab immunotherapy: a case report & review of the literature. *J. Immunother. Cancer* **2016**, *4*, 94, https://doi.org/10.1186/s40425-016-0199-9.
41. Rodriguez, E.F.; Lipson, E.; Suresh, K.; Cappelli, L.C.; Monaco, S.E.; Maleki, Z.. Immune checkpoint block-er-related sarcoid-like granulomatous inflammation: a rare adverse event detected in lymph node aspi-ration cytology of patients treated for advanced malignant melanoma. *Hum Pathol*. **2019**, *91*, 69–76.
42. Laroche, A.; Alarcon, C.E.; Bourgeault, E.; Dore, M.A.. Erythema Nodosum as the Initial Presen-tation of Nivolumab-Induced Sarcoidosis-Like Reaction. *J Cutan Med Surg*. **2018**, *22(6)*, 627–629.
43. Tan, I.; Malinzak, M.; Salama, A.K.S.. Delayed onset of neurosarcoidosis after concurrent ipilimumab/nivolumab therapy. *J Immunother Cancer*. **2018**, *6(1)*, 77.
44. Dunn-Pirio, A.M.; Shah, S.; Eckstein, C. Neurosarcoidosis following Immune Checkpoint Inhibition. *Case Rep. Oncol.* **2018**, *11*, 521–526, https://doi.org/10.1159/000491599.
45. Tulbah, R.I.; Rowe, S.P.; Solnes, L.B.; Javadi, M.S. Nivolumab-Associated Pulmonary and Bone Sarcoidosis in a Patient With Melanoma of Unknown Primary. *Clin. Nucl. Med.* **2019**, *44*, e519–e521, https://doi.org/10.1097/rlu.0000000000002724.
46. Fukuchi, K.; Hikawa, M.; Sano, Y.; Kasuya, A.; Aoshima, M.; Tatsuno, K.. Sarcoid-like reaction and viti-ligo occurring after nivolumab therapy in a patient with metastatic melanoma. *J Dermatol.* **2019**, *46(10)*, e359–e360.
47. Lise, Q.-K.; Audrey, A.-G. Multifocal choroiditis as the first sign of systemic sarcoidosis associated with pembrolizumab. *Am. J. Ophthalmol. Case Rep.* **2016**, *5*, 92–93, https://doi.org/10.1016/j.ajoc.2016.12.014.
48. Nishino, M.; Sholl, L.M.; Awad, M.M.; Hatabu, H.; Armand, P.; Hodi, F.S.. Sarcoid-Like Granulomatosis of the Lung Related to Immune-Checkpoint Inhibitors: Distinct Clinical and Imaging Features of a Unique Im-mune-Related Adverse Event. *Cancer Immunol Res.* **2018**, *6(6)*, 630–635.
49. Smith, R.J.; Mitchell, T.C.; Chu, E.Y. Pembrolizumab-induced sarcoidal infusion site reaction. *J. Cutan. Pathol.* **2018**, *45*, 727–729, https://doi.org/10.1111/cup.13307.
50. Wang, L.; Patel, G.; Chiesa-Fuxench, Z.C.; Mcgettigan, S.; Schuchter, L.; Mitchell, T.C.; Ming, M.E.; Chu, E.Y. Timing of Onset of Adverse Cutaneous Reactions Associated With Programmed Cell Death Protein 1 Inhibitor Therapy. *JAMA Dermatol.* **2018**, *154*, 1057–1061, https://doi.org/10.1001/jamadermatol.2018.1912.
51. Woodbeck, R.; Metelitsa, A.I.; Naert, K.A.. Granulomatous Tumoral Melanosis Associated With Pembrolizumab Therapy: A Mimicker of Disease Progression in Metastatic Melanoma. *Am J Dermatopathol.* **2018**,*40(7)*,523–526.
52. Lu, Y. FDG PET/CT Course of Pembrolizumab-Associated Multiorgan Sarcoidosis. *Clin. Nucl. Med.* **2019**, *44*, 167–168, https://doi.org/10.1097/rlu.0000000000002408.
53. Marcoval, J.; Bauer-Alonso, A.; Fornons-Servent, R.; Jiménez-Colomo, L.; Sabaté-Llobera, A.; Penín, R. Subcutaneous sarcoidosis induced by pembrolizumab in a melanoma patient mimicking subcutaneous metastasis at 18F-FDG PET/CT. **2020**, *40*, 255–256, https://doi.org/10.1016/j.remnie.2020.09.004.
54. Garanzini, E.M.; Scaramuzza, D.; Spadarella, G.; Di Guardo, L.; Marchianò, A. Sarcoidosis-like disease mimicking metastases during adjuvant ipilimumab therapy in advanced melanoma patient: CT scan and MRI help in managing difficult clinical decision. *BJR|case Rep.* **2020**, *6*, https://doi.org/10.1259/bjrcr.20190065.
55. Cervantes, J.; Rosen, A.; Dehesa, L.; Dickinson, G.; Alonso-Llamazares, J.. Granulomatous Reaction in a Patient With Metastatic Melanoma Treated With Ipilimumab: First Case Reported With Isolated Cuta-neous Findings. *Actas Dermosifiliogr (Engl Ed).* **2019**,*110(1)*,43–49.
56. Yasin, H.; Yadala, V.; Khan, N.A.J.; Graffeo, V.; Denning, K.; Lebowicz, Y.. Immunotherapy-Induced Sar-coid-Like Reaction: A Shrewd Imitator. *J Investig Med High Impact Case Rep.* **2021**,*9*,23247096211009400.
